# Washed microbiota transplantation improves patients with metabolic syndrome in South China

**DOI:** 10.3389/fcimb.2022.1044957

**Published:** 2022-11-15

**Authors:** Lei Wu, Xin-Jian Lu, De-Jiang Lin, Wen-Jia Chen, Xing-Ying Xue, Tao Liu, Jia-Ting Xu, Ya-Ting Xie, Man-Qing Li, Wen-Ying Lin, Qing Zhang, Qing-Ping Wu, Xing-Xiang He

**Affiliations:** ^1^ Department of Gastroenterology, Research Center for Engineering Techniques of Microbiota-Targeted Therapies of Guangdong Province, The First Affiliated Hospital of Guangdong Pharmaceutical University, Guangzhou, China; ^2^ Guangdong Provincial Key Laboratory of Microbial Safety and Health, State Key Laboratory of Applied Microbiology Southern China, Institute of Microbiology, Guangdong Academy of Sciences, Guangzhou, China; ^3^ School of Biology and Biological Engineering, South China University of Technology, Guangzhou, China; ^4^ Xiamen Treatgut Biotechnology Co., Ltd., Xiamen, China

**Keywords:** fecal microbiota transplantation (FMT), washed microbiota transplantation (WMT), metabolic syndrome, comprehensive efficacy, atherosclerotic cardiovascular disease (ASCVD)

## Abstract

**Background:**

Metabolic syndrome (MS) is a growing public health problem worldwide. The clinical impact of fecal microbiota transplantation (FMT) from healthy donors in MS patients is unclear, especially in southern Chinese populations. This study aimed to investigate the effect of washed microbiota transplantation (WMT) in MS patients in southern China.

**Methods:**

The clinical data of patients with different indications receiving 1-3 courses of WMT were retrospectively collected. The changes of BMI, blood glucose, blood lipids, blood pressure and other indicators before and after WMT were compared, such as fasting blood glucose (FBG), glycated hemoglobin (HbA1c), total cholesterol (TC), triglyceride (TG), low-density lipoprotein cholesterol (LDL-c)), high-density lipoprotein cholesterol (HDL-c), non-high-density lipoprotein (non-HDL-c), systolic blood pressure (SBP), diastolic blood pressure (DBP), etc. At the same time, comprehensive efficacy evaluation and atherosclerotic cardiovascular disease (ASCVD) grade assessment were performed on MS patients. Finally, 16S rRNA gene amplicon sequencing was performed on fecal samples of MS patients before and after transplantation.

**Results:**

A total of 237 patients were included, including 42 in the MS group and 195 in the non-MS group. For MS patients, WMT significantly improved the comprehensive efficacy of MS in short term 40.48% (p<0.001), medium term 36.00% (p=0.003), and long term 46.15% (p=0.020). Short-term significantly reduced FBG (p=0.023), TG (p=0.030), SBP (p=0.026) and BMI (p=0.031), and increased HDL-c (p=0.036). The medium term had a significant reduction in FBG (p=0.048), TC (p=0.022), LDL-c (p=0.043), non-HDL-c (p=0.024) and BMI (p=0.048). WMT had a significant short term (p=0.029) and medium term (p=0.011) ASCVD downgrading effect in the high-risk group of MS patients. WMT improved gut microbiota in MS patients.

**Conclusion:**

WMT had a significant improvement effect on MS patients and a significant downgrade effect on ASCVD risk in the high-risk group of patients with MS. WMT could restore gut microbiota homeostasis in MS patients. Therefore, the regulation of gut microbiota by WMT may provide a new clinical approach for the treatment of MS.

## Introduction

Metabolic syndrome (MS) has become one of the major public health challenges worldwide. It is a group of risk factors combined with obesity, hyperglycemia (diabetes or impaired glucose regulation), dyslipidemia (hyperglycemia and/or low HDL-c hyperemia), and hypertension of clinical syndromes (O’Neill and O’Driscoll, 2015; Rossi et al., 2022). These conditions co-occur in individuals more frequently than expected by chance. When these factors are combined, they directly contribute to the development of ASCVD and also increase the risk of developing type 2 diabetes (Grundy, 2016; Guembe et al., 2022; Tang et al., 2022). There has been a dramatic increase in the number of people living with MS worldwide, and this increase has been associated with a global epidemic of obesity and diabetes (Grundy, 2008; Simmons et al., 2010; Gurka et al., 2017). With the increased risk of diabetes and cardiovascular disease associated with MS (Gami et al., 2007; DeBoer et al., 2015), strategies to prevent emerging global epidemics are urgently needed.

MS combines multiple symptoms, such as obesity, dyslipidemia, hyperglycemia, and hypertension, which significantly increase the risk, progression rate, and harm of diabetes and cardiovascular disease. Therefore, a scientific and reasonable treatment strategy for MS should be comprehensive, including measures such as blood glucose, blood lipid, blood pressure, weight control, and lifestyle improvement. These treatment strategies are mainly lifestyle intervention and drug therapy. Previous studies have confirmed that exercise has both alleviating and therapeutic effects in improving abnormal glucose and lipid metabolism. For people who are sedentary, current recommendations are to gradually increase aerobic exercise, and moderate physical activity can reduce the risk of metabolic diseases (Donnelly et al., 2009). A meta-analysis of 48 studies reported significant improvements in lipid metabolism in 2990 subjects with MS who underwent moderate-intensity aerobic exercise training (40 to 60% of heart rate reserve or maximal oxygen uptake) (Wood et al., 2022). Various drugs are used to treat MS, such as hypoglycemic agents: metformin (Ludvik et al., 2021), alpha-glucosidase inhibitors (Krentz et al., 2008), targeting the glucagon-like peptide GLP -1 receptor agonists, etc. (Rosenstock et al., 2021; Sattar et al., 2021). Lipid-lowering drugs: statins (Briguori et al., 2009; Di Sciascio et al., 2009; Ray et al., 2021), ezetimibe, etc. (Kim et al., 2022). Antihypertensive drugs: diuretic antihypertensives (Bakris et al., 2012), sympathetic inhibitor (Azizi et al., 2015), calcium antagonists (Carey et al., 2018), renin-angiotensin system inhibitor et al. (Bobrie et al., 2012). Diet pills: orlistat (Dombrowski et al., 2014), topiramate (Yanovski and Yanovski, 2014), liraglutide (Bray et al., 2016), lorcaserin (Gadde et al., 2018). However, long term use of drug therapy can have significant side effects. Therefore, it is of great significance to comprehensively analyze the related factors of MS and find a treatment method with less side effects.

The human gut microbiota has an average of 10-100 trillion microorganisms, more than ten times the estimated number of human cells (Turnbaugh et al., 2007). The impact of a healthy and diverse gut microbiota on metabolic system, immune system, and gut homeostasis is increasingly evident (Jung et al., 2015). The gut microbiome is increasingly recognized as playing an important role in human physiology and health. Gut dysbiosis, defined as a decrease in bacterial diversity or a change in bacterial species compared to healthy controls, is associated with the development of multiple diseases (Machiels et al., 2014; Cani, 2017). Modulation of the gut microbiota to restore a balanced and diverse microbiota may be of great value for the treatment or prevention of microbiome-related diseases. Altering the composition of the gut microbiota has received attention as a novel therapeutic modality to improve insulin sensitivity (Khan et al., 2014). Fecal microbiota transplantation (FMT) is a novel therapeutic approach that uses healthy microbial profiles to replace the patient’s own disturbed microbiota (Bafeta et al., 2017). FMT has a tendency not only to improve the function of commensal host bacteria, but also to completely reshape the entire host microbiome. FMT does this by altering the actual composition and proportions of the resident symbiotic species present in the host. FMT is now included in guidelines recommending FMT as the standard of care in the setting of recurrent *Clostridium difficile* infection (Varier et al., 2015). FMT is of increasing interest (de Groot et al., 2020). FMT is now being tested in clinical trials for other diseases, such as inflammatory bowel disease (IBD) (Moayyedi et al., 2015; Paramsothy et al., 2017; Qazi et al., 2017), Crohn’s disease (Paramsothy et al., 2017), obesity (Allegretti et al., 2020), and functional gastrointestinal disorders (Pamer, 2014), which are also associated with marked dysbiosis. Whether FMT can improve MS is a topic to be explored in clinical medicine.

Washed microbiota transplantation (WMT) is a microbiota transplantation method that is similar to traditional FMT but adds the safety measure of washed microbiota. The biggest difference between WMT and FMT is that the bacterial solution of WMT is prepared by an intelligent microorganism separation system (GenFMTer), which has gone through a multi-level filtration system, and finally the washed bacterial solution of WMT is obtained after several washed. It has better safety, quality control for bacterial flora disorders and effectiveness (Shi, 2020; Zhang et al., 2020). We tried to investigate whether WMT could improve patients with MS in patients with functional bowel disease and other diseases who received WMT in the Department of Gastroenterology, The First Affiliated Hospital of Guangdong Pharmaceutical University. We hypothesized that WMT could safely and consistently affect patients across various indications, improve MS without side effects. Therefore, we conducted a retrospective trial to collect medical data from patients with MS treated with WMT.

## Materials and methods

### Patients and experimental design

This study included patients who received WMT for functional bowel disease and other diseases in our hospital from December 2016 to May 2022 and completed 1-3 courses of treatment. Inclusion criteria: patients older than 18 years who volunteered to receive WMT. Exclusion criteria were: pregnant women, patients taking probiotics during WMT treatment. In the end, a total of 237 people met the requirements. This study was approved by the Ethics Committee of the First Affiliated Hospital of Guangdong Pharmaceutical University, Guangzhou, China according to the Declaration of Helsinki (no. 2017-98). Written informed consent was obtained and reviewed from all patients.

The diagnostic criteria for metabolic syndrome in this study refer to the Chinese Guidelines for the Prevention and Treatment of Type 2 Diabetes (2020 Edition) (Chinese Diabetes S, 2021) and the diagnostic criteria for metabolic syndrome of the Diabetes Society of the Chinese Medical Association (Metabolic Syndrome Research Collaboration Group of Diabetes Branch of ChineseMedical Association, 2018). Metabolic syndrome group (MS group) can be diagnosed with 3 or more of the following: (1) BMI ≥ 25kg/m^2^. (2) Hyperglycemia: fasting blood glucose ≥ 6.1 mmol/L or 2-hour blood glucose after glucose load ≥ 7.8 mmol/L and (or) those who have been diagnosed with diabetes and treated. (3) Hypertension: blood pressure ≥130/85 mmHg (1 mmHg=0.133 kPa) and (or) confirmed hypertension and treated. (4) Fasting triglyceride (TG) ≥ 1.70mmol/L. (5) Fasting HDL-c < 1.04mmol/L. Those who do not meet the above conditions are the non-metabolic syndrome group (non-MS group). Finally, 42 people in the MS group and 195 people in the non-MS group were included. With reference to the Chinese Guidelines for the Prevention of Cardiovascular Diseases (2017 edition) (“Chinese Guidelines for the Prevention of Cardiovascular Diseases (2017),” 2018), the ASCVD risk was assessed according to baseline and blood lipid status.

### Preparation of washed Microbiota and WMT procedure

The procedure of WMT complies with the Nanjing Consensus on the Methodology of Washed Microbiota Transplantation (Shi, 2020). All healthy fecal donors between the ages of 18 and 25 undergo rigorous consultation, psychological and physical examination, biochemical testing and infectious disease screening. To prepare the washed microbiota, each 100 g of feces and 500 mL of 0.9% saline was used to prepare a homogeneous fecal suspension. Then, the washed bacteria solution was prepared by an intelligent microorganism separation system (GenFMTer) (one-hour FMT protocol with relatively low oxygen environment) (Zhang et al., 2020). According to each patient’s physical condition and wishes, the washed bacteria solution is injected into the patient’s body through the upper gastrointestinal tract (nasojejunal tube) or the lower gastrointestinal tract (endoscopic intestinal tube). The center implements the standard of “three three courses of treatment” of WMT. That was, do a WMT course every month for the first three months, and then do a WMT course three months apart after the third month. Among them, a WMT course of 3 days, once a day, once a 120mL of the washed bacteria solution was used to patients (Mullish et al., 2018). The results of blood tests and other tests before the first course of treatment were the baseline values, and relevant indicators were obtained before each subsequent course of treatment. Time could be divided into short term: about 1 month after the first WMT course; medium term: about 2 months after the first WMT course; long term: about 6 months after the first WMT course. All patients underwent at least 2 WMT procedures and completed follow-up.

### Clinical data collection

Baseline values, short, medium, and long term outcomes of patients before treatment were collected. Data included age (year), sex n (%), BMI (kg/m^2^), disease or indication for WMT, laboratory test results. Mainly include blood glucose indicators: fasting blood glucose (FBG, mmol/L), glycosylated hemoglobin (HbA1c, %). Insulin index: fasting insulin (FI, μU/mL), and calculate the insulin resistance value (HOMA-IR, insulin resistance value=fasting blood glucose*fasting insulin/22.5). Blood lipid indexes: total cholesterol (TC, mmol/L), triglyceride (TG, mmol/L), low density lipoprotein cholesterol (LDL-c, mmol/L), high density lipoprotein cholesterol (HDL-c, mmol/L), Apolipoprotein B (ApoB, g/L), non-high density lipoprotein (non-HDL-c, mmol/L), lipoprotein a (LIP, mmol/L). Blood pressure indicators: systolic blood pressure (SBP, mmHg) on admission, diastolic blood pressure (DBP, mmHg) on admission. Adverse events (AEs): abdominal pain, diarrhea, nausea and vomiting, dizziness, fatigue, etc. After all patients received WMT treatment and completed follow-up, the results of blood glucose, blood lipids and blood pressure were statistically analyzed and evaluated.

### DNA extraction and sequencing

Stool samples from 5 patients with MS, 6 patients with non-MS, and 5 donors were collected for sequencing before and after WMT. All samples were stored at -80°C after collection until DNA extraction. DNA extraction from fecal samples was performed as previously described (Qin et al., 2012). DNA quality and concentration were checked by NanoDrop™ 2000 (Thermo Fisher Scientific, Wilmington, DE, USA). A bacterial 16S rRNA gene fragment (V3-V4) was amplified from the extracted DNA by PCR using primers 338F (5′-ACTCCTACGGGAGGCAGCAG-3′) and 806R (5′-GGACTACHVGGGTWTCTAAT-3′). Polymerase chain reaction (PCR) conditions: 30 s at 95°C, 30 s at 55°C, and 45 s at 72°C for a total of 25 cycles. PCR products were subjected to agarose gel electrophoresis to determine the size of the amplicon. The constructed library was quantified by Qubit and Q-PCR; after the library was qualified, the NovaSeq6000 (Illumina, San Diego, CA, USA) sequencing platform was used for on-board sequencing.

### Amplicon data processing and analysis

The sample data was split from the off-machine data, and the barcode and primer sequences were truncated. FLASH (V1.2.11, http://ccb.jhu.edu/software/FLASH/) (Magoc and Salzberg, 2011) software was used to splicing the reads of the sample to obtain Raw Tags. Then, using fastp(0.19.6) (Chen et al., 2018) software to conduct quality control on the obtained Raw Tags to obtain high-quality Clean Tags. Finally, the Clean Tags are compared with the database to detect and remove chimeras (Haas et al., 2011), so as to obtain the final effective data, namely Effective Tags. Using the DADA2 module (Callahan et al., 2016) in the QIIME2 (version 2020.2) (Bolyen et al., 2019) software, and filtering out sequences with an abundance of less than 5, the final ASVs (Amplicon Sequence Variants) and features sheet were obtained. Subsequently, the obtained ASVs were compared with the database using the classify-sklearn module in the QIIME2 software to obtain the species information of each ASV.

### Data analysis

Statistical analysis was performed using SPSS 22.0 (IBM Corp., Armonk, NY, USA) and Prism 8 (GraphPad, San Diego, CA, USA). Results were expressed as frequencies and percentages for categorical variables, mean and standard deviation for continuous variables with a normal distribution. Categorical variables were analyzed using chi-square or Fisher’s exact test. For comparison of continuous variables between two independent groups, an unpaired Student’s-t test (normally distributed variables) could be used. Paired data were compared using paired Student’s-t test (normally distributed variables). Two-tailed p-values < 0.05 were considered statistically significant.

## Results

### Clinical characteristics of patients undergoing WMT

**Figure 1:**
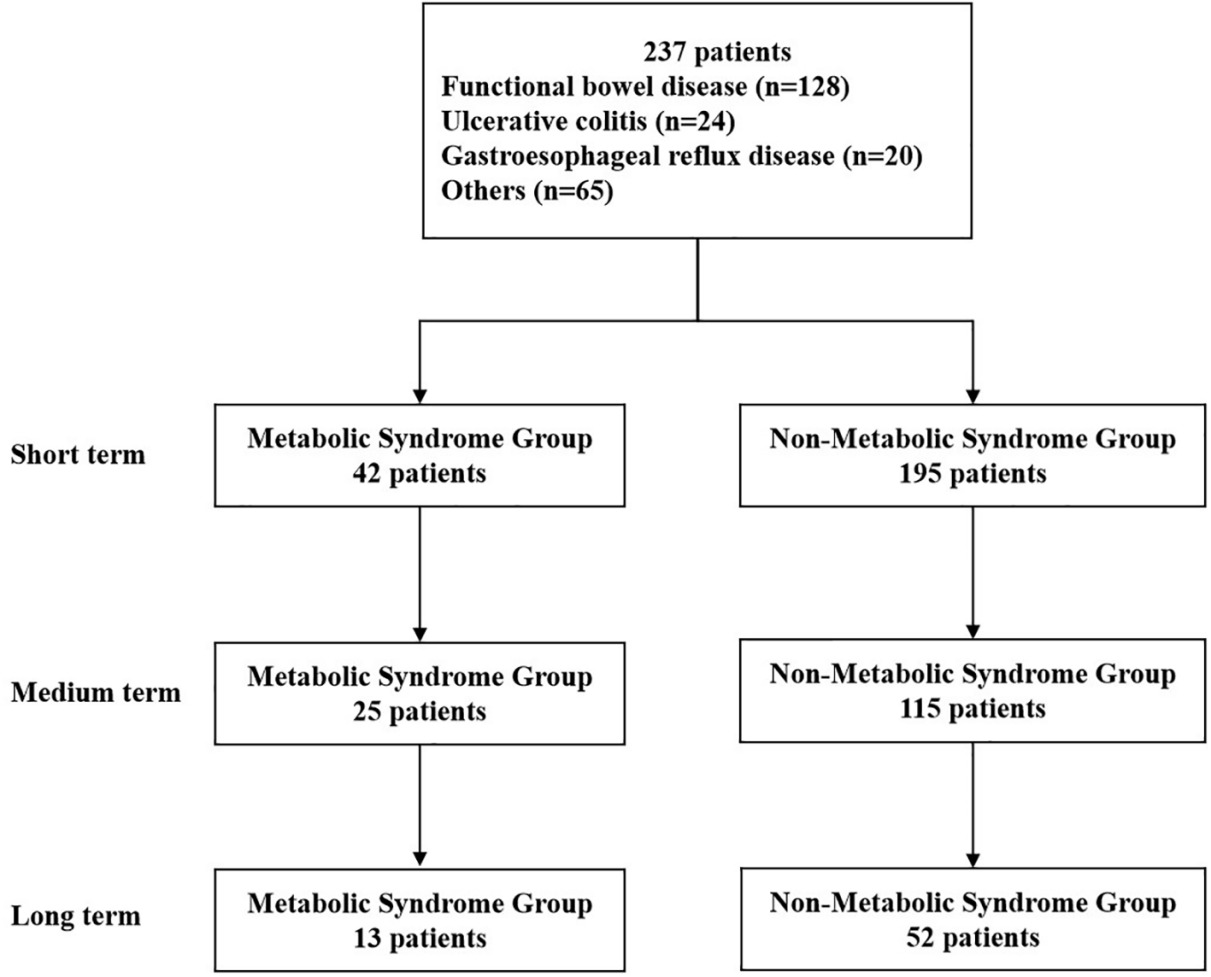
Flow chart of this study. Short term: about 1 month; medium term: about 2 months; long term: about 6 months.

WMT was completed in the First Affiliated Hospital of Guangdong Pharmaceutical University from December 2016 to May 2022. A total of 237 patients (42 in the MS group and 195 in the non-MS group) met the inclusion criteria. Among them, 121 (51.05%) were male, 116 (48.95%) were female, and the median age was 55 (41-63) years old. The analysis process is shown in **Figure 1**. **Table 1** shows the first six disease characteristics of patients undergoing WMT, which are functional bowel disease (n=128, 54.01%, including irritable bowel syndrome, functional constipation), ulcerative colitis (n=24, 10.13%), gastroesophageal reflux disease (n=20, 8.44%), non-alcoholic fatty liver disease (n=11, 4.64%), gouty arthritis (n=7, 2.95%), chemotherapy-related diarrhea (n=7, 2.95%). Due to the different compliance of patients, WMT treatment may not be completed on schedule. In this study, the time interval of WMT in the enrolled patients was counted, and the number of days was expressed as the median (25%-75%). The test results of the patient before the first course of treatment are the baseline value. The second course of treatment was separated by 35 days from baseline (32-42, short term). The third course of treatment was 77 days away from baseline (67-98.75, medium term). The fourth course of treatment was 185 days away from baseline (147.50-210, long term).

**Table 1:** The main diagnoses of patients receiving washed microbiota transplantation.

Primary cause of WMT	Number (n)	Percentage (%)
Functional bowel disease	128	54.01%
Ulcerative colitis	24	10.13%
Gastroesophageal reflux disease	20	8.44%
Nonalcoholic fatty liver	11	4.64%
Gouty arthritis	7	2.95%
Chemotherapy-Associated Diarrhea	7	2.95%
Atopic dermatitis	6	2.53%
Hyperlipidemia	5	2.11%
Post-hepatitis cirrhosis	5	2.11%
Radiation enteritis	5	2.11%
Crohn’s disease	4	1.69%
Hyperlipidemic pancreatitis	2	0.84%
Senile tremor	1	0.42%
Duodenal stasis	1	0.42%
Chronic urticaria	1	0.42%
Parkinson’s syndrome	1	0.42%
Bipolar disorder	1	0.42%
Psoriasis vulgaris	1	0.42%
Hyperuricemia	1	0.42%
Perianal eczema	1	0.42%
Autoimmune hepatitis	1	0.42%
Pustular psoriasis	1	0.42%
Neuromyelitis optica	1	0.42%
Eepression	1	0.42%
Functional dysphagia	1	0.42%
total	237	100.00%

The demographic and clinical characteristics of the patients in the MS group and the non-MS group are compared in **Table 2**. Due to different compliance, not all patients have complete data, so the number of patients in each group is different for each index. There was no significant difference in age and gender ratio between the MS group and the non-MS group, indicating that the basic conditions of the study population were not significantly different, which reduced the confounding factors of this study. BMI (27.56 ± 4.69 vs 21.62 ± 3.41 kg/m^2^, p<0.001), FBG (6.00 ± 1.97 vs 4.80 ± 1.07 mmol/L, p<0.001), HbA1c (6.80 ± 1.22 vs 5.84 ± 0.88%, p=0.007), FI (12.67 ± 8.22 vs 8.27 ± 5.57 μU/mL, p=0.011), HOMA-IR (3.44 ± 2.43 vs 1.86 ± 1.68, p=0.003), TC (5.43 ± 2.04 vs 4.69 ± 1.07 mmol/L, p=0.027), TG (3.85 ± 4.72 vs 1.06 ± 0.54 mmol/L, p<0.001), HDL-c (1.00 ± 0.27 vs 1.36 ± 0.31 mmol/L, p<0.001), ApoB (1.07 ± 0.31 vs 0.89 ± 0.24 g/L, p<0.001), non-HDL-c (4.41 ± 2.09 vs 3.33 ± 1.01 mmol/L, p=0.002), SBP (132.57 ± 11.66 vs 120.85 ± 13.94 mmHg, p<0.001), DBP (82.62 ± 10.72 vs 76.50 ± 9.59 mmHg, p<0.001), the above indexes in the MS group were significantly higher than those in the non-MS group except for HDL-c (mmol/L).

**Table 2:** Demographics and clinical characteristics of patients at baseline.

	Metabolic syndrome group (42)	Non-metabolic syndrome group (195)	Donors (5)
Age (year)	54.98 ± 14.53 (n=42)	52.02 ± 15.81 (n=195)	22.80 ± 0.84 (n=5)
Male n (%)	54.76	50.26	80.00
BMI (kg/m^2^)	27.56 ± 4.69 (n=38)	21.62 ± 3.41 (n=190)	21.26 ± 1.23 (n=5)
FBG (mmol/L)	6.00 ± 1.97 (n=42)	4.80 ± 1.07 (n=193)	4.60 ± 0.24 (n=5)
HbA1c (%)	6.80 ± 1.22 (n=19)	5.84 ± 0.88 (n=21)	/
FI (μU/mL)	12.67 ± 8.22 (n=28)	8.27 ± 5.57 (n=105)	/
HOMA-IR	3.44 ± 2.43 (n=28)	1.86 ± 1.68 (n=102)	/
TC (mmol/L)	5.43 ± 2.04 (n=42)	4.69 ± 1.07 (n=167)	0.79 ± 0.36 (n=5)
TG (mmol/L)	3.85 ± 4.72 (n=42)	1.06 ± 0.54 (n=167)	2.32 ± 1.21 (n=5)
LDL-c (mmol/L)	2.77 ± 0.98 (n=42)	2.85 ± 0.96 (n=167)	1.25 ± 0.36 (n=5)
HDL-c (mmol/L)	1.00 ± 0.27 (n=42)	1.36 ± 0.31 (n=167)	0.79 ± 0.36 (n=5)
ApoB (g/L)	1.07 ± 0.31 (n=42)	0.89 ± 0.24 (n=167)	/
non-HDL-c (mmol/L)	4.41 ± 2.09 (n=42)	3.33 ± 1.01 (n=167)	2.90 ± 0.29 (n=5)
LIP (mmol/L)	118.16 ± 158.69 (n=19)	139.13 ± 138.18 (n=50)	/
SBP (mmHg)	132.57 ± 11.66 (n=42)	120.85 ± 13.94 (n=195)	122.40 ± 10.64 (n=5)
DBP (mmHg)	82.62 ± 10.72 (n=42)	76.50 ± 9.59 (n=195)	74.00 ± 7.97 (n=5)

Data presented as mean ± standard deviation, or n (%).

BMI (kg/m^2^), Body mass index; FBG (mmol/L), Fasting blood glucose; HbA1c (%), Glycated hemoglobin; FI (μU/mL), Fasting insulin; HOMA-IR, Homeostasis model assessment of insulin resistance; TC (mmol/L), Total cholesterol; TG (mmol/L), Triglyceride; LDL-c (mmol/L), Low-density lipoprotein cholesterol; HDL-c (mmol/L), High-density lipoprotein cholesterol; ApoB (g/L), Apolipoprotein B; non-HDL-c (mmol/L), Non-HDL cholesterol; LIP (mmol/L), Lipoprotein; SBP (mmHg), Systolic blood pressure; DBP (mmHg), Diastolic blood pressure.

### Evaluation of clinical comprehensive curative effect of WMT on metabolic syndrome

All enrolled patients were divided into MS group and non-MS group according to the evaluation criteria of MS. Patients were regrouped according to changes in MS symptoms after WMT treatment (**Table 3**). The comprehensive curative effect of the patients in the MS group changed significantly during the treatment. Short term recovery was 40.48% (p < 0.001), medium term recovery was 36.00% (p = 0.003), and long term recovery was 46.15% (p = 0.020). Our data suggest that WMT has short, medium, and long term significant metabolic syndrome-improving efficacy in patients with MS; however, the efficacy of WMT remains to be explored. There were very few patients in the non-MS group who increased during WMT treatment, but there is no statistical difference, which may be caused by changes in living habits and other factors during the treatment process for as short as one month or as long as six months. Our data show that WMT has a significant overall improvement in MS.

**Table 3:** Comprehensive clinical efficacy of short, medium and long term treatment on MS.

Data periods	Before therapy (n)	Therapeutic effect base on diagnostic level of MS			
		Unchangedgroup (n)	Changedgroup (n, %)	X^2^	p-Value
**MS group**					
Short term	42	25	17 (40.48%)	21.313	<0.001
Medium term	25	16	9 (36.00%)	8.672	0.003
Long term	13	7	6 (46.15%)	5.417	0.020
**Non-MS group**					
Short term	195	191	4 (2.05%)	2.273	0.132
Medium term	115	110	5 (4.35%)	3.271	0.071
Long term	52	50	2 (3.85%)	0.510	0.475

The definition of unchanged and changed of MS group were still MS group and changed to Non-MS group.

The definition of unchanged and changed of Non-MS group were still Non-MS and changed to MS group.

### Efficacy evaluation of WMT in the treatment of ASCVD risk

According to ASCVD risk stratification, patients were divided into very high risk group, high risk group, intermediate risk group and low risk group. After WMT treatment, patients were regrouped into risk-modified and risk-modified groups (**Table 4**). Acute coronary syndrome, stable coronary heart disease, ischemic cardiomyopathy, ischemic stroke, transient ischemic attack, and peripheral atherosclerosis were included in the very high-risk group. This group of patients was not reassigned after WMT treatment and is not listed in **Table 4**.

**Table 4:** Effects of short, medium, and long term treatment on atherosclerotic cardiovascular disease risk classification of patients with MS.

Data periods	Before therapy (n)	Therapeutic effect base on ASCVD risk stratification			
		Unchangedgroup (n)	Risk descended (n, %)	X^2^	p-Value
High-risk group					
Short term	24	18	6 (25.0%)	4.762	0.029
Medium term	17	10	7 (41.2%)	6.476	0.011
Long term	8	4	4 (50.0%)	3.000	0.083
Medium-risk group					
Short term	10	6	4 (40.0%)	2.813	0.094
Medium term	7	3	4 (57.1%)	3.150	0.076
Long term	4	3	1 (25.0%)	0.000	1.000

The definition of Risk descended of high- and medium-risk groups were medium-, and low-risk after WMT procedures, respectively.

In terms of ASCVD rating, in the high-risk group of patients with MS, WMT in the short term and medium term had a significant effect, with 25.0% (p=0.029) in the short term and 41.2% (p=0.011) in the medium-risk group. There was no statistically significant effect of short term, medium term and long term WMT on ASCVD rating in the medium-risk group. In conclusion, in terms of ASCVD rating, WMT has a significant short and medium term ASCVD downgrading effect in the high-risk group of patients with MS.

### Comparative analysis of each index after WMT treatment and baseline

**Figure 2:**
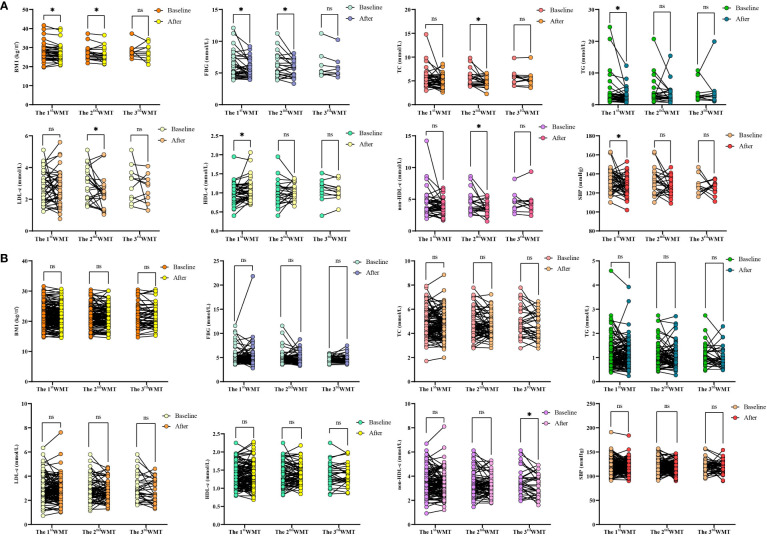
Changes of BMI、FBG、TC、TG、LDL-c、HDL-c、non-HDL-c and SBP levels after 1-3 times of WMT. **(A)** Changes of BMI、FBG、TC、TG、LDL-c、HDL-c、non-HDL-c and SBP in MS group; **(B)** Changes of BMI、FBG、TC、TG、LDL-c、HDL-c、non-HDL-c and SBP in non-MS group. BMI, Body mass index; FBG, Fasting blood glucose; TC, Total cholesterol; TG, Triglyceride; LDL-c, Low-density lipoprotein cholesterol; HDL-c, High-density lipoprotein cholesterol; non-HDL-c, Non-HDL cholesterol; SBP, Systolic blood pressure. * indicates p < 0.05; ns, not significant. Short term: about 1 month; medium term: about 2 months; long term: about 6 months.

Previously, we observed that WMT had a significant improvement effect on MS as a whole, and then we further analyzed the specific indicators. **Table 5** and **Figure 2** showed the effects of WMT on BMI, blood glucose, blood lipids, and blood pressure in patients with MS. The results showed that in the MS group, WMT had a significantly lower effect (p < 0.05) on BMI in the short term (from 27.56 ± 4.69 to 26.95 ± 4.47kg/m^2^, p = 0.031) and in the medium term (from 27.32 ± 3.56 to 26.46 ± 3.70kg/m^2^, p = 0.048)). And in the long term (from 28.19 ± 3.62 to 27.70 ± 4.24kg/m^2^), it also showed a reducing effect, but because the number of people was too small, it was not significant in the long term (p = 0.767). This suggests that WMT has a good BMI-improving effect in patients with MS. FBG had a significantly lower effect (p < 0.05) in the short term (from 6.03 ± 1.98 to 5.49 ± 1.34 mmol/L, p = 0.023) and in the medium term (from 6.21 ± 1.96 to 5.66 ± 1.28 mmol/L, p = 0.048). And in the long term (from 6.24 ± 2.25 to 5.95 ± 1.89mmol/L), it also showed a reducing effect, but because the number of people was too small, it was not significant in the long-term (p = 0.163). This suggests that WMT has a good blood glucose-improving effect in patients with MS. Meanwhile, in the MS group, WMT significantly reduced TC (from 5.52 ± 1.59 to 4.71 ± 1.02mmol/L, p = 0.022) in the medium term (p < 0.05). TG (from 3.90 ± 4.84 to 2.62 ± 2.19mmol/L, p = 0.030) had a significant short term reduction (p < 0.05). LDL-c (from 2.85 ± 1.04 to 2.36 ± 0.98mmol/L, p = 0.043) was significantly lower in the medium term (p < 0.05). HDL-c (from 0.99 ± 0.27 to 1.07 ± 0.29mmol/L, p = 0.036) had a significant increase in the medium term (p < 0.05). non-HDL-c (from 4.46 ± 1.63 to 3.68 ± 1.05mmol/L, p = 0.024) had a significant lowering effect in the mid-term (p < 0.05). Overall, WMT significantly improved blood lipids in the MS group. There was a significant short term reduction (p < 0.05) on SBP (from 132.57 ± 11.66 to 127.64 ± 10.20mmHg, p = 0.026), indicating that WMT has a significant antihypertensive effect on blood pressure in patients with MS. In the non-MS group, WMT had a significant long term reduction (p < 0.05) on non-HDL-c (from 3.54 ± 1.06 to 3.24 ± 0.94mmol/L, p = 0.033), however, there was no significant change in FBG, TC, TG, LDL-c, HDL-c in the short term, medium term and long term, that is, WMT had no significant change in the non-MS group.

**Table 5:** The comparison values of each index in high MS group and non-MS group in the short term, medium term and long term with baseline during the treatment of washed microbiota transplantation.

Items	Baseline	Short term	p-Value	Baseline	Medium term	p-Value	Baseline	Long term	p-Value
**MS group**									
BMI (kg/m^2^)	27.56 ± 4.69 (n=38)	26.95 ± 4.47 (n=38)	0.031	27.32 ± 3.56 (n=21)	26.46 ± 3.70 (n=21)	0.048	28.19 ± 3.62 (n=10)	27.70 ± 4.24 (n=10)	0.767
FBG (mmol/L)	6.03 ± 1.98 (n=38)	5.49 ± 1.34 (n=38)	0.023	6.21 ± 1.96 (n=22)	5.66 ± 1.28 (n=22)	0.048	6.24 ± 2.25 (n=8)	5.95 ± 1.89 (n=8)	0.163
HbA1c (%)	7.06 ± 0.78 (n=8)	7.05 ± 0.93 (n=8)	0.949	7.92 ± 0.97 (n=5)	7.50 ± 0.54 (n=5)	0.184	8.55 ± 1.34 (n=2)	7.65 ± 0.64 (n=2)	0.323
FI (μU/mL)	13.83 ± 7.73 (n=20)	14.14 ± 6.37 (n=20)	0.863	12.51 ± 6.40 (n=10)	13.55 ± 6.89 (n=10)	0.662	13.94 ± 6.41 (n=8)	15.28 ± 6.42 (n=8)	0.529
HOMA-IR	3.72 ± 2.35 (n=20)	3.70 ± 2.11 (n=20)	0.971	3.74 ± 1.81 (n=10)	4.08 ± 2.64 (n=10)	0.729	4.00 ± 2.01 (n=7)	4.06 ± 2.01 (n=7)	0.931
TC (mmol/L)	5.42 ± 2.08 (n=40)	4.91 ± 1.26 (n=40)	0.125	5.52 ± 1.59 (n=24)	4.71 ± 1.02 (n=24)	0.022	5.77 ± 1.62 (n=11)	5.47 ± 1.67 (n=11)	0.235
TG (mmol/L)	3.90 ± 4.84 (n=40)	2.62 ± 2.19 (n=40)	0.030	4.10 ± 4.34 (n=24)	3.35 ± 3.11 (n=24)	0.410	3.89 ± 3.15 (n=11)	4.18 ± 5.32 (n=11)	0.820
LDL-c (mmol/L)	2.75 ± 1.00 (n=40)	2.71 ± 1.09 (n=40)	0.836	2.85 ± 1.04 (n=24)	2.36 ± 0.98 (n=24)	0.043	3.01 ± 1.11 (n=11)	2.63 ± 0.94 (n=11)	0.328
HDL-c (mmol/L)	0.99 ± 0.27 (n=40)	1.07 ± 0.29 (n=40)	0.036	1.00 ± 0.33 (n=24)	1.03 ± 0.20 (n=24)	0.608	1.08 ± 0.29 (n=11)	1.05 ± 0.24 (n=11)	0.253
ApoB (g/L)	1.07 ± 0.31 (n=40)	1.02 ± 0.26 (n=40)	0.369	1.13 ± 0.31 (n=24)	0.99 ± 0.25 (n=24)	0.060	1.19 ± 0.35 (n=11)	1.15 ± 0.20 (n=11)	0.627
non-HDL-c (mmol/L)	4.40 ± 2.15 (n=40)	3.84 ± 1.18 (n=40)	0.085	4.46 ± 1.63 (n=24)	3.68 ± 1.05 (n=24)	0.024	4.58 ± 1.50 (n=11)	4.43 ± 1.80 (n=11)	0.591
LIP (mmol/L)	139.67 ± 178.64 (n=14)	153.49 ± 204.34 (n=14)	0.254	69.02 ± 118.45 (n=6)	71.05 ± 105.31 (n=6)	0.807	/	/	/
SBP (mmHg)	132.57 ± 11.66 (n=42)	127.64 ± 10.20 (n=42)	0.026	131.72 ± 13.51 (n=25)	126.40 ± 9.12 (n=25)	0.070	127.08 ± 9.50 (n=12)	125.17 ± 7.22 (n=12)	0.628
DBP (mmHg)	82.62 ± 10.72 (n=42)	78.43 ± 9.13 (n=42)	0.051	81.76 ± 11.18 (n=25)	79.40 ± 7.44 (n=25)	0.323	81.00 ± 9.20 (n=12)	83.08 ± 8.59 (n=12)	0.536
Non-MS group									
BMI (kg/m^2^)	21.62 ± 3.42 (n=187)	21.50 ± 3.32 (n=187)	0.279	21.53 ± 3.47 (n=112)	21.30 ± 3.36 (n=112)	0.206	21.69 ± 3.90 (n=51)	21.76 ± 3.66 (n=51)	0.776
FBG (mmol/L)	4.80 ± 1.10 (n=170)	4.72 ± 1.62 (n=170)	0.405	4.77 ± 1.11 (n=98)	4.64 ± 0.90 (n=98)	0.207	4.63 ± 0.59 (n=47)	4.66 ± 0.79 (n=47)	0.775
HbA1c (%)	5.43 ± 1.37 (n=4)	5.45 ± 1.10 (n=4)	0.895	/	/	/	/	/	/
FI (μU/mL)	8.71 ± 6.33 (n=61)	8.53 ± 5.17 (n=61)	0.728	9.78 ± 7.52 (n=32)	10.39 ± 7.56 (n=32)	0.489	10.66 ± 5.67 (n=16)	9.53 ± 4.74 (n=16)	0.299
HOMA-IR	2.02 ± 2.01 (n=60)	1.95 ± 1.64 (n=60)	0.651	2.27 ± 2.50 (n=32)	2.36 ± 2.14 (n=32)	0.699	2.19 ± 1.33 (n=16)	1.99 ± 1.13 (n=16)	0.377
TC (mmol/L)	4.76 ± 1.12 (n=116)	4.67 ± 1.07 (n=116)	0.225	4.72 ± 1.09 (n=70)	4.60 ± 0.90 (n=70)	0.277	4.92 ± 1.21 (n=31)	4.62 ± 1.07 (n=31)	0.051
TG (mmol/L)	1.15 ± 0.59 (n=116)	1.09 ± 0.54 (n=116)	0.129	1.08 ± 0.49 (n=70)	1.03 ± 0.48 (n=70)	0.405	1.12 ± 0.47 (n=31)	1.00 ± 0.43 (n=31)	0.164
LDL-c (mmol/L)	2.90 ± 0.99 (n=116)	2.84 ± 0.96 (n=116)	0.370	2.84 ± 0.94 (n=70)	2.76 ± 0.79 (n=70)	0.352	3.03 ± 1.04 (n=31)	2.78 ± 0.92 (n=31)	0.080
HDL-c (mmol/L)	1.34 ± 0.30 (n=116)	1.33 ± 0.33 (n=116)	0.623	1.38 ± 0.32 (n=70)	1.37 ± 0.28 (n=70)	0.692	1.38 ± 0.31 (n=31)	1.38 ± 0.29 (n=31)	0.982
ApoB (g/L)	0.90 ± 0.25 (n=116)	0.90 ± 0.26 (n=116)	0.874	0.89 ± 0.23 (n=70)	0.89 ± 0.23 (n=70)	0.946	0.93 ± 0.22 (n=31)	0.93 ± 0.24 (n=31)	0.927
non-HDL-c (mmol/L)	3.42 ± 1.05 (n=116)	3.34 ± 1.03 (n=116)	0.255	3.34 ± 1.00 (n=70)	3.23 ± 0.84 (n=70)	0.271	3.54 ± 1.06 (n=31)	3.24 ± 0.94 (n=31)	0.033
LIP (mmol/L)	114.58 ± 93.83 (n=22)	122.48 ± 102.74 (n=22)	0.243	119.23 ± 88.80 (n=12)	124.18 ± 112.83 (n=12)	0.732	87.13 ± 49.56 (n=3)	75.30 ± 56.66 (n=3)	0.442
SBP (mmHg)	120.85 ± 13.94 (n=195)	119.44 ± 12.2 (n=195)	0.164	120.75 ± 13.42 (n=115)	119.20 ± 12.57 (n=115)	0.306	120.57 ± 12.73 (n=51)	119.47 ± 12.03 (n=51)	0.597
DBP (mmHg)	76.5 ± 9.59 (n=195)	75.97 ± 9.05 (n=195)	0.500	76.21 ± 9.54 (n=115)	74.37 ± 8.26 (n=115)	0.082	75.69 ± 8.79 (n=51)	75.96 ± 7.68 (n=51)	0.841

Data presented as mean ± standard deviation, or n (%).

### Correlation analysis of WMT on indicators affecting MS

**Figure 3:**
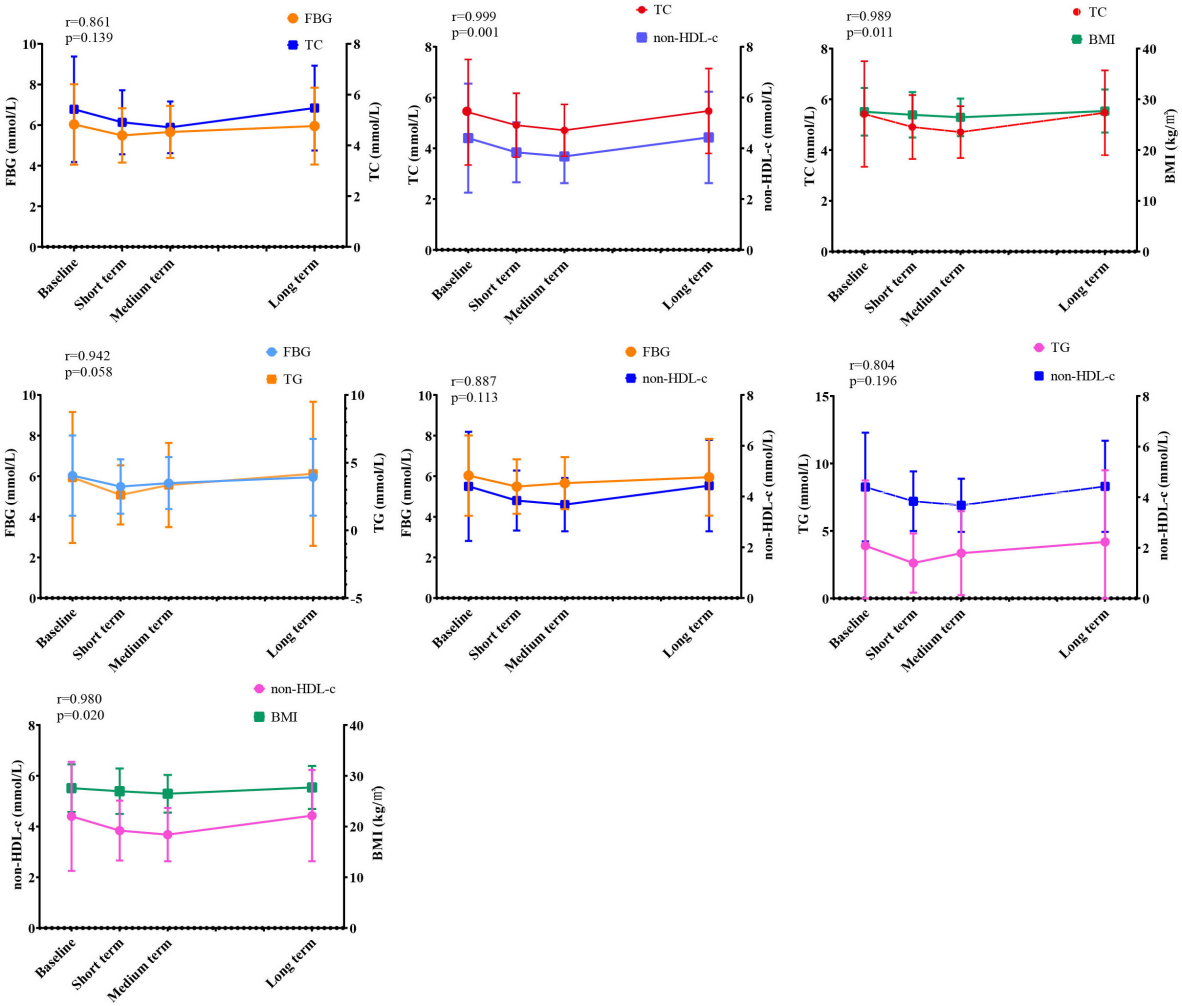
Correlation analysis of WMT on MS regulation. BMI, Body mass index; TC, Total cholesterol; FBG, Fasting blood glucose; non-HDL-c, Non-HDL cholesterol; TG, Triglyceride. (r≦0.3, indicating poor correlation; 0.3<r≦0.6, indicating moderately strong correlation; 0.6<r≦0.8, indicating strong correlation; r>0.8, indicating extremely strong correlation). Short term: about 1 month; medium term: about 2 months; long term: about 6 months.

We previously found that WMT significantly improved BMI, FBG, TC, TG, LDL-c, HDL-c, non-HDL-c and SBP in the MS group during treatment. In order to find the relevant factors affecting the regulation of WMT on MS, correlation analysis was performed on the above-mentioned indicators with significant regulating effect. As shown in **Figure 3**, we found that in the MS group, TC was strongly positively correlated with FBG, non-HDL-c, and BMI. FBG was strongly positively correlated with TG and non-HDL-c. There was a strong positive correlation between non-HDL-c and TG and BMI. Our data show that in the process of WMT treatment, while improving BMI, blood glucose and blood lipids are also affected by a good improvement, and BMI, blood glucose, and blood lipids have a strong correlation. This provides us with a good therapeutic idea for the treatment of MS. That is to say, WMT has a significant effect on the treatment MS. It can also play a role in weight loss and lipid lowering while reducing blood glucose, and at the same time, it plays a comprehensive role in these three aspects.

### Analysis of the composition of gut microbiota before and after WMT

**Figure 4:**
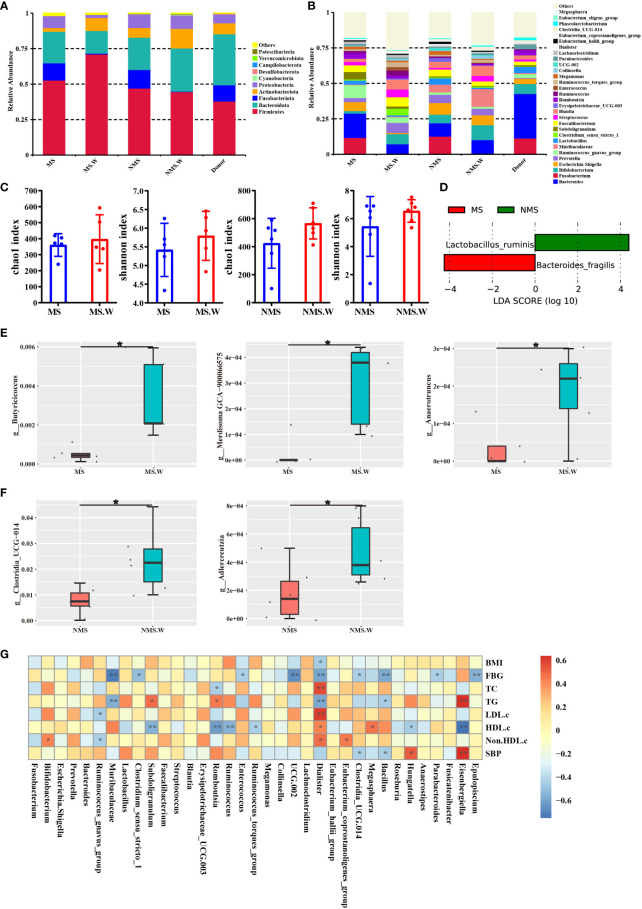
The composition of gut microbiota before and after WMT. **(A)** Composition of the top ten gut microbiota at the phylum level. **(B)** Composition of the top 30 gut microbiota at the genus level. **(C)** Chao1 index and Shannon index of alpha diversity analysis. **(D)** LEfSe analysis of MS group and non-MS group. **(E)** T-test analysis of MS group before and after WMT. **(F)** T-test analysis of non-MS group before and after WMT. **(G)** Mutual variation relationship between environmental factors and species. MS: In MS group before WMT. MS.W: In MS group after WMT. NMS: In non-MS group before WMT. NMS.W: In non-MS group after WMT. BMI, Body mass index; FBG, Fasting blood glucose; TC, Total cholesterol; TG, Triglyceride; LDL-c, Low-density lipoprotein cholesterol; HDL-c, High-density lipoprotein cholesterol; non-HDL-c, Non-HDL cholesterol; SBP, Systolic blood pressure. * indicates p < 0.05; ** indicates p < 0.01. Short term: about 1 month; medium term: about 2 months; long term: about 6 months.

We analyzed the gut microbiota composition of the MS group, the non-MS group, and donors before and after WMT. The gut microbiota at the phylum level mainly includes *Firmicutes, Bacteroidota, Fusobacteriota, Actinobacteriota and Proteobacteria*. For the MS group, at the phylum level, WMT increased the relative abundance of *Firmicutes* and *Actinobacteriota*; decreased the relative abundance of *Fusobacteriota* and *Proteobacteria* (**Figure 4A**). At the class level, the relative abundances of *Clostridia, Bacilli, Actinobacteria*, and *Coriobacteriia* were increased after WMT; the relative abundances of *Fusobacteriia, Bacteroidia, Gammaproteobacteria*, and *Negativicutes* were decreased after WMT (**Supplementary Figure 1A**). At the order level, the relative abundances of *Lachnospirales, Bifidobacteriales, Oscillospirales, Lactobacillales, Clostridiales* and *Erysipelotrichales* were increased after WMT; the relative abundances of *Fusobacteriales, Bacteroidales, Enterobacterales* and *Peptostreptococcales Tissierellales* were decreased after WMT (**Supplementary Figure 1B**). At the family level, the relative abundances of *Lachnospiraceae, Bifidobacteriaceae, Prevotellaceae, Ruminococcaceae* and *Clostridiaceae* were increased after WMT; the relative abundances of *Fusobacteriaceae, Bacteroidaceae, Enterobacteriaceae*, etc. were decreased after WMT (**Supplementary Figure 1C**). At the genus level, the relative abundances of *Bifidobacterium, Prevotella, Muribaculaceae, Clostridium, Faecalibacterium, Streptococcus*, and *Blautia* were increased after WMT; the relative abundances of *Fusobacterium, Bacteroides, Escherichia-Shigella, Ruminococcus*, etc. were decreased after WMT (**Figure 4B**). Among them, WMT can not only increase the relative abundance of beneficial bacteria, such as *Bifidobacterium*, *Prevotella*, *Faecalibacterium*, etc. In addition, it can reduce the relative abundance of harmful bacteria, such as poisonous *Fusobacterium*, poisonous *Bacteroides*, *Escherichia-Shigella* and so on. WMT could increase the α-diversity of gut microbiota in both the MS group and the non-MS group, with an increased chao1 index indicating an increase in the total number of community species, and an increased shannon index indicating an increase in community species diversity (**Figure 4C**). We performed LEfSe analysis on the MS group and the non-MS group in order to find the Biomarker with statistical difference between the two groups. Finally, it was found that the differential species in the MS group was *Bacteroides fragilis*, and the differential species in the non-MS group was *Lactobacillus ruminis* (**Figure 4D**). Species with significant differences between the MS groups before and after WMT were identified by T-test. We found that WMT significantly increased the relative abundances of *Butyricicoccus*, *Merdisoma*, and *Anaerotruncus* at the genus level of gut microbiota in the MS group compared with baseline (**Figure 4E**). Meanwhile, WMT significantly increased the relative abundance of *Clostridia* and *Adlercreutzia* at the genus level of the gut microbiota in the non-MS group (**Figure 4F**). We used Spearman rank correlation to study the mutual change relationship between environmental factors and species, and obtained the correlation and significant P value between the two groups. We found a significant negative correlation between BMI and *Dialister*. FBG was significantly negatively correlated with *Epulopiscium, Parabacteroides, Bacillus, Clostridia, Dialister, Enterococcus, Clostridium* and *Muribaculaceae*. TC was significantly negatively correlated with *Romboutsia*. TG was significantly negatively correlated with *Bacillus*, *Dialister* and *Muribaculaceae*. LDL.c was significantly negatively correlated with *Ruminococcus*. HDL.c was significantly positively correlated with *Megasphaera* and *Eubacterium*. Non.HDL.c was significantly negatively correlated with *Ruminococcus*. SBP was significantly negatively correlated with *Bacillus* and *Clostridia* (**Figure 4G**).

### Prevalence of AEs in WMT patients

We also analyzed the prevalence of AEs in patients receiving WMT. WMT-related AEs were determined on the basis of clinical judgment and all available information (primarily diarrhea, abdominal pain, nausea, vomiting, generalized joint pain, fatigue, convulsions, rash, fever, and dizziness). A total of 679 WMT procedures were analyzed, and the overall incidence of AEs was 2.50%. Diarrhea was the most common AE (7 cases, 1.03%), followed by abdominal pain (2 cases, 0.29%), nausea and vomiting (2 cases, 0.29%), generalized joint pain (1 case, 0.15%), fatigue (1 case, 0.15%), convulsions (1 case, 0.15%), rash (1 case, 0.15%), fever (1 case, 0.15%) and dizziness (1 case, 0.15%). The reality is that these effects quickly go away on their own and pose no greater threat to the patient’s health.

## Discussion

WMT has a significant improvement effect in patients with MS and a significant downgrade effect on ASCVD risk. In terms of comprehensive efficacy, WMT has significantly improved curative effect in short, medium and long term in patients with MS. In terms of ASCVD rating, WMT has a significant short and medium term ASCVD downgrading effect in the high-risk group of patients with MS. WMT can significantly improve blood glucose, blood lipids, blood pressure and BMI in patients with MS. Our data suggest that modulation of the gut microbiota by WMT may be a novel approach for the treatment of MS.

The gut microbial community is known to be critical for processing dietary polysaccharides, promoting the absorption of monosaccharides in the gut, and inducing hepatic lipogenesis. The gut microbiota also influenced the energy and energy stores that the host obtains from the diet (Turnbaugh and Gordon, 2009). Numerous experiments in animal models or humans showed that FMT played an important role in body weight regulation (Ridaura et al., 2013). In a FMT study of patients with MS, the recipient gut microbiota after transplantation was similar to that of the donor (Li et al., 2016). Donor-specific microorganisms *Roseburia hominis*, *Ruminococcus lactaris* and *A. muciniphila* were able to successfully colonize the recipient (Azad et al., 2018), the latter being associated with improved host glucose tolerance. These results suggested that improving gut dysbiosis with FMT may be an effective treatment for obesity. Specifically, SCFA-producing bacteria, such as *Roseburia gutis*, *Bryantella forexigens*, and *Megamonas hypermegale*, were significantly increased after FMT (Smits et al., 2018), which may help improve insulin sensitivity in patients with MS. Next Lai et al. found that transplantation of fecal microbiota from mice on a normal-fat diet into mice on a high-fat diet significantly reduced appetitive efficacy, body weight, and metabolic profile in mice on a high-fat diet. The beneficial effects of linking exercise to a normal diet could be transmitted through FMT, and FMT could improve inflammatory status and metabolism in obesity. The transmissible beneficial effects of FMT had been explained by overexpression of *Odoribacter* and oxidative phosphorylation and glycolysis genes (Lai et al., 2018). Consistently, our results showed a similar effect in patients with MS. That was, WMT had a significant short term and medium term improvement effect on BMI in patients with MS. This may be related to the increased abundance of beneficial bacterial species after transplantation. WMT could lead to an increase in the proportion of *Firmicutes* in the recipients as described in the text and in the **Figure 4A**. It seems reasonable to interpret that the MS group itself contained a high abundance of *Firmicutes*, while receiving *Firmicutes* from donors, resulting in a logical increase in the relative abundance of *Firmicutes*. Similarly, similar results were observed in the non-MS group. Of course, the increased of *Firmicutes* was not a direct addition of numbers. It was possible that the gut microbiota showed up in an interacted form after WMT. *A. muciniphila* was one such species commonly associated with anti-obesity traits. For example, in mouse and a few human studies, *A. muciniphila* significantly improved body composition and nutrient processing in obese subjects. *A. muciniphila* was underrepresented in the microbiome of a mouse model of type 2 diabetes (Okubo et al., 2018). The ratio of *Firmicutes* to *Bacteroidetes* was often used when correlating changes in microbiota composition with obesity phenotypes. *Firmicutes* had been documented to be more abundant than *Bacteroidetes* in obese subjects, whereas lean individuals had more *Bacteroidetes* and less *Firmicutes* (Furet et al., 2010; Louis et al., 2016).

Scientific reports showed that FMT could also improve plasma metabolic parameters in patients with MS. For example, Vrieze et al. found improvements in peripheral and hepatic insulin sensitivity six weeks after infusion of microbiota from lean donors into recipients with MS, and concluded that the gut microbiota could potentially be developed as a therapeutic agent to increase insulin sensitivity in humans (Vrieze et al., 2012). Next, Kootte et al. investigated the effects of lean donor (allogeneic) versus own (autologous) fecal microbiota transplantation in male recipients with MS. Similar to the team of Vrieze et al., at 6 weeks after FMT, the authors observed improved peripheral insulin sensitivity, elevated postprandial plasma triglycerides, and reduced glycated hemoglobin (HbA1c) levels in recipient plasma obtained from allogeneic donors (Kootte et al., 2017). A similarly interesting experiment was performed by Sung et al. The authors administered fecal microbiota to obese mice by oral gavage in their experiments from resveratrol-fed and normally fed groups of donor mice. Their findings showed improved glucose clearance in the group of mice that received fecal suspensions from resveratrol-fed donors compared to the group of normally fed donor mice (Sung et al., 2017). Consistently, our results showed a similar effect in patients with MS. It was shown that WMT had a significant improving effect on blood glucose in patients with MS. WMT had a significant effect on reducing fasting blood glucose in the short and medium term in patients with MS. These data leaded us to conclude that the delivery of beneficial microbiota or metabolites *via* FMT could have ameliorating effects on glycemia in patients with MS.

Hyperlipidemia (HLP) was considered to be an important risk factor for cardiovascular diseases (CVDs) (Roth et al., 2020), atherosclerosis (Panahi et al., 2018; Libby et al., 2019), diabetes (Sun et al., 2022). Evidence suggested that the gut microbiota played an important role in the regulation of energy metabolism and lipid levels in the host (Velagapudi et al., 2010; Mestdagh et al., 2012). A non-human primate model of hyperlipidemia (HLP) was established in cynomolgus monkeys fed a high-fat diet (HFD) for 19 months, according to a recent study. Transplantation of fecal microbiota from HFD-T (high-fat diet-tolerant) monkeys into HFD rats attenuated HLP and hepatic steatosis (Gao et al., 2022). Probiotics could protect the host from intestinal dysbiosis, thereby conferring health benefits to the host. *Lactobacillus* had been reported to have a lipid-lowering effect on hypercholesterolemic and hyperlipidemic rats or mice (Singh et al., 2015; Qian et al., 2019). *A muciniphila* was currently recommended as a new potential complementary therapy for clinical obesity and diabetes (Plovier et al., 2017; Cani and de Vos, 2017). *P. distasonis* had been shown to have metabolic benefits in reducing body weight, hyperglycemia, and hepatic steatosis in genetically obese (ob/ob) and high-fat diet (HFD)-fed mice (Wang et al., 2019). Furthermore, *Faecalibacterium prausnitzii* and its secreted peptides exhibited anti-inflammatory effects against chemically induced colitis in mice (Breyner et al., 2017). Consistently, our results showed a similar effect in patients with MS. It was shown that WMT had a significant improving effect on blood lipids in patients with MS. WMT could significantly reduce triglyceride and increase high-density lipoprotein in patients with MS in the short term. In the medium term, it could significantly reduce total cholesterol, low-density lipoprotein, and non-high-density lipoprotein. Therefore, it is promising to ameliorate such diseases and gut dysbiosis by targeting the modulation of gut microbiota using probiotics or FMT.

Several intervention studies had shown that blood pressure in animal models of hypertension can be altered by altering the gut microbiota. Yang et al. observed a significant decrease in microbial richness, diversity, and uniformity in spontaneously hypertensive rats, and an increase in the ratio of *Firmicutes*/*Bacteroidetes*. These changes were accompanied by a decrease in acetic and butyric acid-producing bacteria (Yang et al., 2015). Furthermore, the microbiota of a small group of human hypertensive patients was found to follow a similar pattern of dysbiosis. Yang et al. discovered a role for the brain-gut-kidney axis in maintaining normal homeostasis and dysregulation of this axis in chronic kidney disease and hypertension may lead to new therapeutic targets (Yang et al., 2018). In addition, high fiber and SCFA-acetate supplementation had been reported to alter gut microbiota, increase the abundance of acetate-producing bacteria, and prevent hyperactivity in a mouse model of deoxycorticosterone acetate (DOCA)-induced hypertension. Blood pressure (Marques et al., 2017). Another meta-analysis on the antihypertensive effect of probiotics showed that multiple probiotics had a greater effect on blood pressure improvement than a single probiotic (Khalesi et al., 2014). As a multi-species gut microbiota transplant, FMT had significant antihypertensive effects in hypertensive animals (Toral et al., 2019). Consistently, our results showed similar effects in patients with MS. However, our study showed that WMT had a clinically significant short term blood pressure lowering effect, with a trend to lower blood pressure in the medium and long term, but the effect was not significant. Although the significant antihypertensive effect of WMT was short term, it also had a trend of antihypertensive in the medium and long term, and the effect of WMT was generally longer than that of traditional antihypertensive drugs. Further studies are needed to explore how to prolong the antihypertensive effect of WMT. Several clinical studies had found that the gut microbiota of hypertensive patients differs significantly from that of healthy controls, characterized by loss of microbial diversity, loss of beneficial bacteria, and increase in potentially harmful bacteria (Yan et al., 2017; Li et al., 2017). Our 16S rRNA sequencing data suggested that WMT may restore microbial diversity in patients with MS and modulate their microbial composition, similar to that observed in healthy controls. In addition, research by Zhong et al. showed that WMT has antihypertensive effect on hypertensive patients (Zhong et al., 2021). Zhong et al. found that genus-level relative abundance of gut microbiota in hypertensive patients after WMT significantly changed compared with baseline, including increased abundance of *Senegalimassilia* and decreased abundance of *Parasutterella* and *Solobacterium*. Adamberg et al. showed that higher abundance of *Senegalimassilia* was associated with healthy traits. They found that children without obesity had higher levels of *Senegalimassilia anaerobia* compared to overweight children (Adamberg et al., 2018). In addition, diabetic rats treated with formulations that could significantly improve hyperglycemia had also been shown to have a high abundance of *Senegalimassilia* (Gao et al., 2018). In addition, the abundance of *Parasutterella* (increased in hypertensive patients) (Mushtaq et al., 2019) and *Solobacterium* (associated with atherosclerotic cardiovascular disease) (Tierney et al., 2021) were significantly increased after WMT reduce. Consistently, our results showed a similar effect in patients with MS. That was, WMT could significantly improve blood pressure in patients with MS. Therefore, WMT may improve blood pressure in patients with MS by restoring gut microbiota homeostasis.

MS combines multiple symptoms, such as obesity, dyslipidemia, hyperglycemia, and hypertension, which significantly increase the risk, progression rate, and harm of diabetes and cardiovascular disease. Therefore, a scientific and reasonable treatment strategy for MS should be based on the control of blood glucose, blood lipids, blood pressure, and body weight for comprehensive treatment. In our study, WMT significantly improved blood glucose, blood lipids, blood pressure and BMI in patients with MS and had a significant downgrading effect on ASCVD risk. We speculate that the improvement of MS after WMT is due to the improvement of gut microbiota after WMT, which comprehensively regulates blood glucose, blood lipids, blood pressure, and BMI. Although there were several studies on WMT function, such as Liang et al. showed that WMT treatment could alter blood lipids in patients with hyperlipidemia and hypolipidemia without serious adverse events (Liang et al., 2022). Wu et al. showed that WMT can significantly improve blood glucose in patients with high blood glucose (Wu et al., 2022a). Pan et al. showed that WMT significantly improved children with autism spectrum disorder, gastrointestinal symptoms and sleep disturbance, and reduced systemic inflammation (Pan et al., 2022). The mechanism by which FMT contributes to disease remission remains largely unexplained. MS may be alleviated by the synergistic effect of gut commensal microbiota after FMT treatment.

Future research should focus on the bacterial species and functional changes associated with FMT treatment in patients with MS, and how FMT affects the metabolism of other organs in long term improvement. Due to the complexity of the gut microbiota, further studies should explore whether specific microbial community species or communities in FMT are dedicated to the prevention and treatment of MS. This may provide a new perspective and reference. Many factors influenced the outcome of FMT, namely donor selection and preparation, sample handling, mode of administration, and colonization resistance (Leshem et al., 2019). Perhaps the healthy donors from the World Longevity Township in South China can provide a better source of donors for patients in South China (Wu et al., 2021a). FMT-related AEs were a challenge for FMT applications. In most cases, mild gastrointestinal AEs were well tolerated by FMT (Wang et al., 2016). Zhang et al. demonstrated the preparation of washed microflora by repeated centrifugation plus suspension three times on the basis of an automated purification system, which significantly reduced AEs (Zhang et al., 2020). Our WMT project was based on Zhang’s standards. No serious AEs were identified during and after WMT. The current understanding of the effects of WMT on the gut microbiota on metabolic diseases is still in its infancy, and data on the effects of WMT on MS are lacking. This is a large-scale retrospective trial of MS in southern China, including a MS group and a non-MS group. The clinical evidence of the effect of WMT on MS has been established, laying a foundation for the follow-up study of the effects of gut microbiota (Wu et al., 2021b) and metabolic markers (Wu et al., 2022b) on metabolic abnormalities. Taken together, these results suggest that restoring gut microbiota may serve as a promising treatment for MS; however, the mechanism of action requires further investigation.

This study had several limitations. First, this study focused on the analysis of clinical BMI, blood glucose metabolism, lipid metabolism, and blood pressure, as well as gut microbiota amplicons before and after WMT. Given that this was a retrospective study, stool samples from patients with MS were rarely collected. WMT could increase the α-diversity of gut microbiota in both the MS group and the non-MS group, with an increased chao1 index indicating an increase in the total number of community species, and an increased shannon index indicating an increase in community species diversity (**Figure 4C**). An important reason for the lack of significant differences here was that our sample size was too small. Because our study was a retrospective study, a large number of samples were not collected before, so the current number of samples was too small to have a significant difference. Our situation was similar to that of Zhong et al. (2021). However, the trend of our results fully agreed with the theoretical results on WMT can reshape the intestinal microecology. Gut microbiota metagenomics and metabolomics before and after WMT had not been evaluated. Therefore, the mechanism of action of WMT in improving ms had not been elucidated. Second, the number of patients and the impact of compliance. The overall number of patients with MS was relatively small, and a small number of patients did not receive long term treatment after short term treatment, and returned to the hospital at short or long intervals to evaluate the long term benefit of WMT treatment. Therefore, more samples and data were needed to confirm the long term efficacy of WMT in the treatment of MS. Third, we did not consider potential confounding factors between the main symptoms of WMT treatment and MS. Based on current limitations, although data showed that WMT could improve MS, the findings of WMT-improving MS should be interpreted with caution, and we need large-scale prospective studies to further validate our conclusions. In the future, we plan to conduct a large-sample prospective study to verify the effect of WMT on MS.

## Conclusions

WMT had a significant improvement in patients with MS and a significant short and medium term downgrading effect on ASCVD risk in the high-risk group of patients with MS. WMT could restore gut microbiota homeostasis in patients with MS. Therefore, the regulation of gut microbiota by WMT may provide a new clinical approach for the treatment of MS.

## Data availability statement

The datasets presented in this study can be found in online repositories. The names of the repository/repositories and accession number(s) can be found below: https://www.ncbi.nlm.nih.gov/, PRJNA881922.

## Ethics statement

The studies involving human participants were reviewed and approved by This study was conducted and approved by the Ethics Committee (No. 2017-98) in accordance with the Declaration of Helsinki at the First Affiliated Hospital of Guangdong Pharmaceutical University, Guangzhou, China. The patients/participants provided their written informed consent to participate in this study.

## Author contributions

X-XH, Q-PW, and LW designed the concept of the study. X-JL, D-JL, W-JC, X-YX, Y-TX, M-QL, and J-TX collected and analyzed the data. TL and W-YL were the statistics consultant, QZ was the consultant for endocrinology. LW wrote the draft manuscript. All authors contributed to the article and approved the submitted version.

## Funding

This study was supported by the Key-Area Research and Development Program of Guangdong Province (No. 2022B1111070006), the Medical Scientific Research Foundation of Guangdong Province (No. B2022209), the Scientific Research Projects of Guangdong Bureau of Traditional Chinese Medicine (No. 20221232), and the Guangdong Innovation Research Team for Higher Education (No. 2021KCXTD025).

## Acknowledgments

We sincerely thank all patients in the study and all funding agencies that supported the study.

## Conflict of interest

Author X-YX was employed by Xiamen Treatgut Biotechnology Co., Ltd., Xiamen, China.

The remaining authors declare that the research was conducted in the absence of any commercial or financial relationships that could be construed as a potential conflict of interest.

## Publisher’s note

All claims expressed in this article are solely those of the authors and do not necessarily represent those of their affiliated organizations, or those of the publisher, the editors and the reviewers. Any product that may be evaluated in this article, or claim that may be made by its manufacturer, is not guaranteed or endorsed by the publisher.

## Supplementary material

Supplementary Figure 1 The composition of gut microbiota before and after WMT at the class, order and family level. **(A)** Composition of the top ten gut microbiota at the class level. **(B)** Composition of the top ten gut microbiota at the order level. **(C)** Composition of the top ten gut microbiota at the family level.
