# Supplementary figures and images for: Washed microbiota transplantation improves patients with metabolic syndrome in South China
Source: Front Cell Infect Microbiol. 2022 Nov 15;12:1044957. doi: 10.3389/fcimb.2022.1044957 (PMC9705737; doi:10.3389/fcimb.2022.1044957)

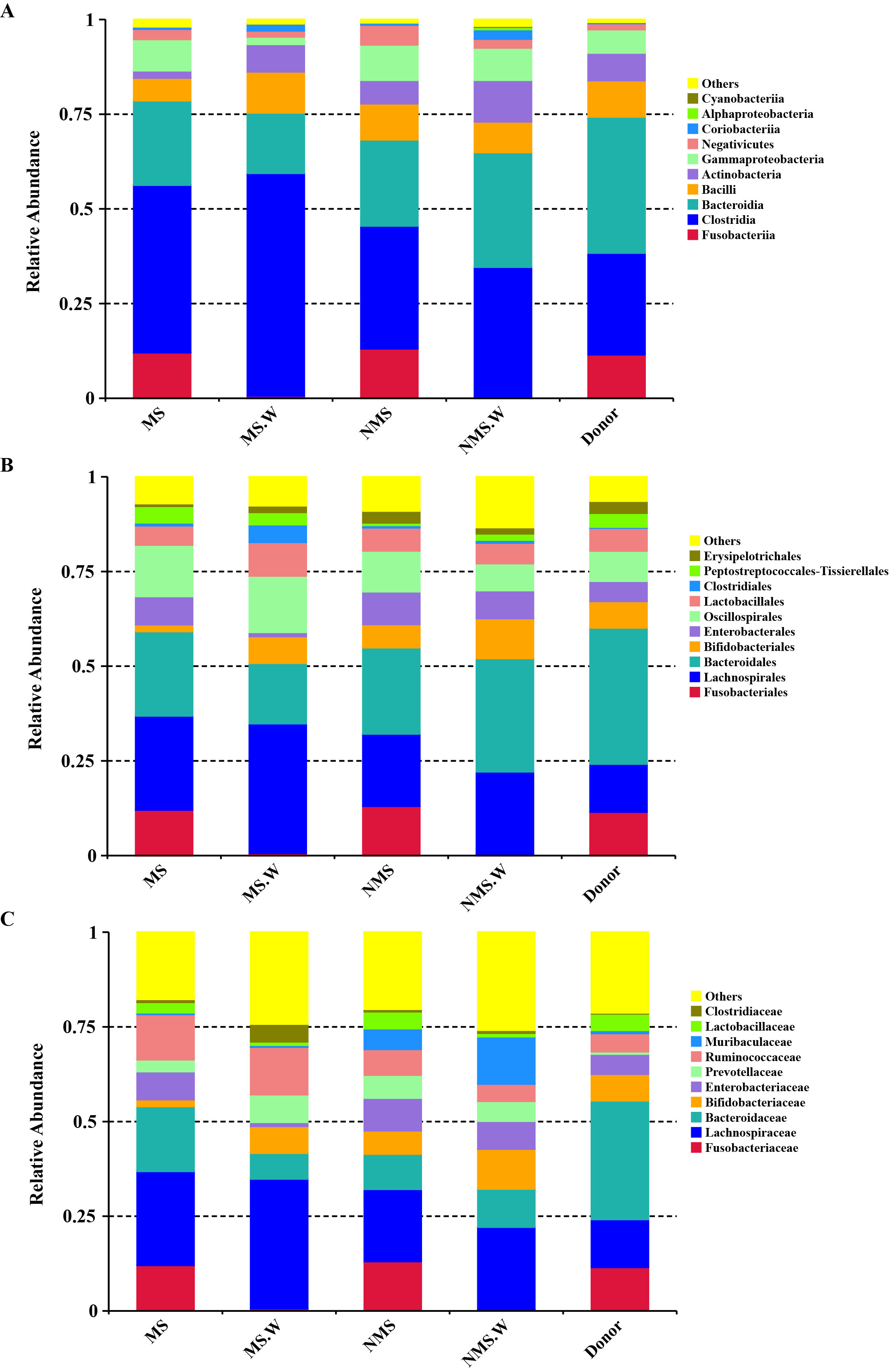

Supplement: Supplementary Figure 1 — The composition of gut microbiota before and after WMT at the class, order and family level. (A) Composition of the top ten gut microbiota at the class level. (B) Composition of the top ten gut microbiota at the order level. (C) Composition of the top ten gut microbiota at the family level. [file Image_1.jpeg]
